# Automated quality assessment of large digitised histology cohorts by artificial intelligence

**DOI:** 10.1038/s41598-022-08351-5

**Published:** 2022-03-23

**Authors:** Maryam Haghighat, Lisa Browning, Korsuk Sirinukunwattana, Stefano Malacrino, Nasullah Khalid Alham, Richard Colling, Ying Cui, Emad Rakha, Freddie C. Hamdy, Clare Verrill, Jens Rittscher

**Affiliations:** 1 Department of Engineering Science, Institute of Biomedical Engineering (IBME), University of Oxford, Oxford, UK; 2 Department of Cellular Pathology, Oxford University Hospitals NHS Foundation Trust, John Radcliffe Hospital, Oxford, UK; 3 NIHR Oxford Biomedical Research Centre, Oxford University Hospitals NHS Foundation Trust, Oxford, Oxfordshire UK; 4 Nuffield Department of Surgical Sciences, University of Oxford, John Radcliffe Hospital, Oxford, UK; 5 School of Medicine, University of Nottingham, Nottingham, UK; 6 CSIRO, Brisbane, QLD Australia

**Keywords:** Image processing, Cancer imaging

## Abstract

Research using whole slide images (WSIs) of histopathology slides has increased exponentially over recent years. Glass slides from retrospective cohorts, some with patient follow-up data are digitised for the development and validation of artificial intelligence (AI) tools. Such resources, therefore, become very important, with the need to ensure that their quality is of the standard necessary for downstream AI development. However, manual quality control of large cohorts of WSIs by visual assessment is unfeasible, and whilst quality control AI algorithms exist, these focus on bespoke aspects of image quality, e.g. focus, or use traditional machine-learning methods, which are unable to classify the range of potential image artefacts that should be considered. In this study, we have trained and validated a multi-task deep neural network to automate the process of quality control of a large retrospective cohort of prostate cases from which glass slides have been scanned several years after production, to determine both the usability of the images at the diagnostic level (considered in this study to be the minimal standard for research) and the common image artefacts present. Using a two-layer approach, quality overlays of WSIs were generated from a quality assessment (QA) undertaken at patch-level at $5\times$ magnification. From these quality overlays the slide-level quality scores were predicted and then compared to those generated by three specialist urological pathologists, with a Pearson correlation of 0.89 for overall ‘usability’ (at a diagnostic level), and 0.87 and 0.82 for focus and H&E staining quality scores respectively. To demonstrate its wider potential utility, we subsequently applied our QA pipeline to the TCGA prostate cancer cohort and to a colorectal cancer cohort, for comparison. Our model, designated as PathProfiler, indicates comparable predicted usability of images from the cohorts assessed (86–90% of WSIs predicted to be usable), and perhaps more significantly is able to predict WSIs that could benefit from an intervention such as re-scanning or re-staining for quality improvement. We have shown in this study that AI can be used to automate the process of quality control of large retrospective WSI cohorts to maximise their utility for research.

## Introduction

Research using digitised histopathology slides (whole slide images; WSIs) for the development of artificial intelligence (AI) algorithms has increased markedly over recent years^1–4^, with pathology having been highlighted as being ‘ripe’ for such innovation in the UK Government’s Industrial Life Sciences Strategy^5^. Whilst such algorithms have the potential to support pathologists diagnostically, thus improving safety, efficiency and quality of reporting, perhaps more significant is their promise in the derivation of novel insights into disease biology and behaviour which are unachievable with a human observer. The impact of AI in histopathology is foreseen by many to be revolutionary with anticipated impact across the whole patient journey from the diagnosis of disease, assessment of prognostic and predictive features, and even stratification of patients for optimal clinical management including allocation to treatment within large clinical trials. The door is being opened to the prospect of deeper understanding of morphomolecular correlations, spatially linking morphology with molecular alterations. Such innovations require big datasets and for some diseases, in order to derive novel prognostic insight, there is the requirement for associated clinical outcome data.

Managing and profiling such large image datasets is time consuming and needs to be automated before such studies can be scaled up. Quality-related issues are recognised to be critical to both the development of AI algorithms and thereafter with their clinical utility and transferability between diagnostic laboratories. This is particularly relevant in relation to WSIs of historic glass slide being collated retrospectively, where artefacts are likely to be more commonly encountered, and to cohorts from multiple diagnostic laboratories with variability in pre-analytical tissue processing and slide storage^6^.

Large retrospective cohorts are a rare commodity, particularly those with accompanying curated clinical data. These then become hugely valuable in the quest for AI-derived predictive features, whether they be morphological, morphogenetic, or biomarker-related; insights which will be truly transformative. This is particularly highlighted in relation to prostate cancer research where the long natural history of the disease poses challenges with only the intermediate and long-term endpoints of metastasis free survival or overall survival being considered valid outcomes^7^. Prostate cancer research exemplifies therefore the ongoing significance of historic or retrospective cohorts to scientific discovery, despite the ability now for diagnostic laboratories with access to digital pathology (DP) to prospectively collate digitised glass slides for research, including algorithm development.

These historic cohorts are often digitised years after the glass slides have been produced and archived, thus introducing the risk of age-related artefacts in relation to the glass slides and subsequently to the quality of the WSI. Indeed it was our own experience of the digitisation of the ProMPT (Prostate Cancer Mechanisms of Progression and Treatment) prostate cancer cohort locally that highlighted to us the impact of age-related factors on WSI quality; the notable artefacts including the presence of H&E staining issues (related to fading and loss of nuclear detail), dirt on the glass, bubble-artefact or loosening of the coverslip due to glue failure, all with the potential to impact image focus and quality.

Consideration of quality issues is also relevant in the context of the already publicly available multi-institutional cohorts, one of the most well-known of which is The Cancer Genome Atlas (TCGA); a cohort example with huge potential in terms of facilitating AI development^8^. Such a huge and varied dataset lends itself to the development of AI tools, and examples of successes to date include ‘decision support’ tools to facilitate tumour classification^9^. However, in spite of these successes, there is a need to remain mindful of the variable nature of the H&E slides present within such cohorts, and therefore also the potential variability in WSI quality. Whilst some datasets may have been curated in terms of a diagnostic ‘label’ associated with a WSI, curation of WSI for quality assurance purposes is not necessarily guaranteed; often it is unclear what quality assurance process has been conducted, if any.

Quality of WSI can be impacted by artefacts present intrinsically within the tissue, by artefacts introduced during slide preparation (including H&E staining) and digitisation (e.g. scanner/focus issues), and potentially by those introduced as a result of ageing and long-term slide storage, the latter of which are encountered in an unpredictable manner in archived slides. Examples include variability in tissue section thickness, tissue folding, H&E staining, air bubbles and/or dirt under the coverslip^10^. Whilst variation in some features may not be such an issue for WSI of glass slides from a single laboratory, and particularly with quality assurance schemes in place for clinical laboratories, such as through the UK Accreditation Service (UKAS), such variation may become more relevant when collating WSIs from multi-institutional cohorts and particularly with older glass slides. Fading of the H&E stain is a recognised feature of slide aging^11^, and numerous authors have investigated methods to overcome the batch effect conferred by this artefact within the research setting, e.g. Vicory et al.^12^. But such artefacts are an equally important consideration in the diagnostic setting and therefore increasingly relevant as laboratories implement digital pathology with pathologists becoming reliant on image quality for accurate diagnosis.

To avoid introducing bias in algorithm development, it is ideal for datasets to be truly representative, encompassing the range of ‘data’ that would be expected in real-life^13^, including both the expected range of (normal and pathological) tissue features and the anticipated variation in tissue and slide preparation between laboratories.

The approach to algorithm development as ‘all inclusive’ using datasets with variation of quality of cases, or by using a more tailored approach with selected high-quality regions/cases will be dependent to an extent upon the research question. In studies investigating potential prognostic features for example, associated patient outcome data is needed and thereby may limit the availability of cases/images for inclusion within the dataset, making appropriate quality of those images critical.

Image quality has been shown to impact on downstream model performance and reliability of results, for example Dodge et al.^14^ have shown that whilst by training models utilizing low quality images may improve test results on low quality images, it can reduce model performance on high quality images. Furthermore, training with increased number of samples leads to longer training times and may increase uncertainty over high-quality samples.

The quality of the WSIs therefore remains an essential consideration during the development and deployment of any algorithm^6^, and is increasingly recognized as such by the broader medical imaging community^15^. Currently this is generally reliant upon manual curation of images being considered for analysis, which is time consuming and inefficient, particularly in the context of a lack of pathologist resource. Furthermore, the reliance upon human observers for QA is perhaps questionable given the recognised subjectivity and poor inter-observer concordance in such tasks, even amongst expert pathologists^16,17^. This issue is further complicated by the interpretation of what is appropriate quality or ‘usable’ which for the expert pathologist is dependent upon the diagnostic question.

It would seem then that an automated method for the assessment of image quality offers the potential to improve efficiency and consistency in such a task. However, QA in relation to histopathology images is complex. Whilst tools exist for image QA of natural scenes^18^, they cannot be directly applied to histological images^19^ due to natural complexity of tissue features and the distinction of artefact types. This is complicated further by the need to determine a threshold for acceptability of the image, which is best defined by an expert, and a means to predict this based on the features assessed.

Perhaps unsurprisingly the availability of QA tools developed specifically for histopathology slides is currently limited^20–26^. Available tools employ traditional hand-crafted features rather than learned ones^23,26^, or tend to be limited to identification of out-of-focus regions only^20–23^ or identification of one artefact per image^24,25^. However, assessment for a combination of artefacts is more meaningful as in real life image artefacts are rarely limited to one feature such as poor staining or tissue folding, particularly with older glass slides. Recognition of the potential for a quality improvement intervention such as re-scanning or re-staining, is more effective if all artefacts present in the image are known; re-scanning will unlikely resolve a quality issue when there are other artefacts such as dirt or ink over the tissue area. Available tools also do not indicate the ’usability’ of the WSI, which is an important parameter when deciding whether a WSI should be included within a cohort for algorithm development.

To address this gap we have developed PathProfiler; an AI tool to automate the QA of WSIs of a retrospective cohort, using the ProMPT prostate cohort as an example. We demonstrate the reliability of PathProfiler in predicting the presence of multiple artefacts in a WSI together with an indication of their impact on image quality, as assessed at the level of usability for clinical diagnosis and highlight the need for collaborative efforts for further development and utility.

PathProfiler provides an image QA at both patch-level and slide-level. Our pipeline generates whole slide quality overlays and predicts the overall usability of each WSI (a value in the range of 0–1 that can be binarised to 0 or 1), and a score 0–10 for quality of focus and H&E staining from the lowest quality to the highest quality.

## Results

### Quality assessment of prostate cancer cohorts

**Figure 1:**
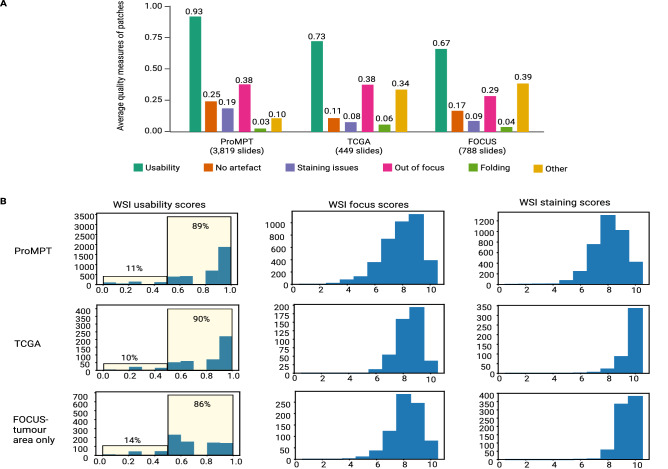
(**A**) Average estimated quality measures of patches extracted from the entire ProMPT cohort (3819 WSIs), and the TCGA-prostate (449 WSIs), and FOCUS (788 WSIs) cohorts. (**B**) Distribution of estimated usability scores of WSIs in each cohort (entire ProMPT cohort, TCGA, and FOCUS) and distribution of estimated scores for WSI focus and H&E staining quality (0–10, lowest quality to highest quality). WSI scores for the FOCUS cohort are calculated for tumour regions only.

We utilised PathProfiler to analyse the entire ProMPT cohort and the 449 WSIs of prostate tissue from TCGA. The breakdown of predicted patch-level artefacts is shown in Fig. 1A. Our model predicts an average usability score of 0.93 and 0.73 for image patches within tissue area in the ProMPT and in the TCGA prostate cohort respectively. The main quality impacting artefacts in the ProMPT cohort are predicted to be related to focus and staining issues, with an average patch score of 0.38 for focus quality issues and an average patch score of 0.19 for staining quality issues (patch scores are between 0 and 1).

Image patches extracted from TCGA slides are highly associated with ‘other’ artefacts, affecting the usability of the slides in comparison with the ProMPT cohort (average patch score of 0.34 vs 0.1). On direct visualisation of the TCGA WSIs it is seen that many of the ‘unusable’ regions are related to the presence of ‘ink’ applied to the glass slide (prior to scanning) by pathologists to highlight areas of interest on the glass slide and which are retained on the WSI. Ink artefact by comparison is rarely seen in the ProMPT and contemporary prostate biopsy cohorts as the glass slides were cleaned of ink to enable the scanner to focus on the tissue rather than the ink.

Predicted slide-level quality scores are shown in Fig. 1B. Our model predicts that 11% of WSIs in the ProMPT cohort are unusable. Whilst we have defined the threshold for usability at the subjective level of that regarded as minimum standard for diagnostic interpretation, the threshold for the cut-off means that a slight shift of predicted score impacts significantly on the binary ‘usable’ vs ‘unusable’ and perhaps it is better to regard the term ‘unusable’ as the cut off at which an image is flagged for manual quality review. According to our criteria, 10% of WSIs in TCGA are estimated to be unusable. In addition, at least 2% of slides in ProMPT are predicted to benefit from re-scanning (84 slides) while at least 0.45% are predicted to benefit from re-staining (17 slides). These values are reported based on the cut-off threshold ($<=4$) for focus and staining quality scores. However, our investigations of the pathologists’ annotations show that many slides annotated as ‘unusable’ with quality scores of 5 or 6 are still suggested for a re-scan/re-stain to solve the quality problem. Therefore, from the distribution of WSI focus and staining scores shown in Fig. 1B, we can conclude that most of ‘unusable’ slides in the ProMPT cohort would benefit from either re-scanning or re-staining.

#### Patch-level quality assessment for selected ProMPT and contemporary WSI cohorts

**Figure 2:**
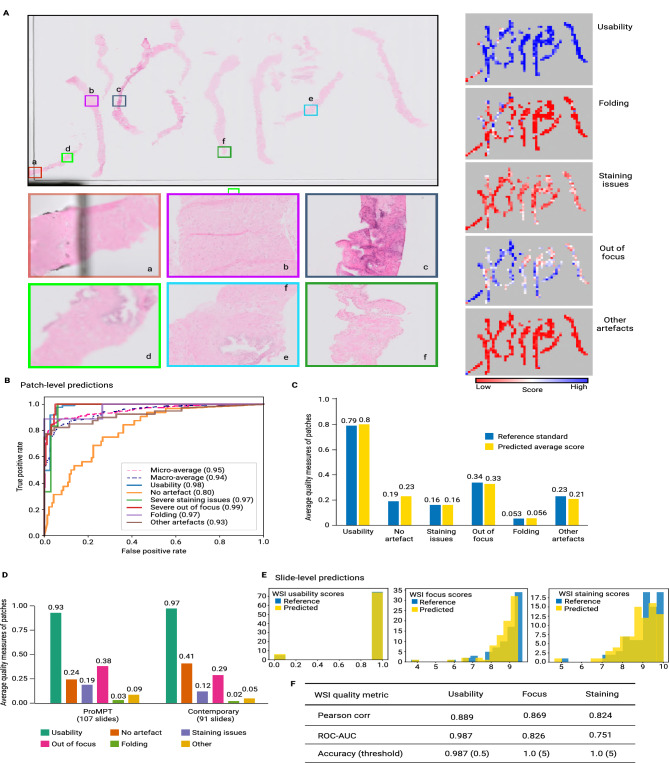
(**A**) H&E WSI (top left) and predicted quality overlays (heatmaps, right). The predicted usability overlay suggests that most image patches are usable. The individual artefact heatmaps indicate patches predicted to show the various artefacts, e.g., a = ‘other’ artefact, in this case coverslip edge and unusable, b & c = folded tissue (although the probability of b being usable is higher than c), d & e = severe focus issue predicted, affecting usability of d but without predicted impact on usability of e, f = slight H&E staining issue predicted (again with minimal impact on usability). Predictions like these could be used in an image analysis pipeline or be made available to a pathologist for regional investigation of artefacts. (**B**, **C**) Model performance for test dataset of image patches (combined ProMPT/contemporary): (**B**) ROC-AUC curves for each category of artefacts and overall usability, and (**C**) average predicted quality measures versus the reference standard as assessed by the pathologist. (**D**) Average predicted quality measures of all image patches in the ‘selected’ ProMPT slides (107 slides) and contemporary archive (91 slides). Such summarised patch-level QA of images provides general cohort-level quality metrics for computational pathology. (**E**) Distribution of ‘reference standard’ vs ‘predicted’ slide-level usability and quality scores for focus and H&E staining (0–10, low to high quality) for the test dataset of image slides (combined ProMPT/contemporary). (**F**) ROC-AUC and Pearson correlation coefficient of the slide-level predictions for usability and quality scores (0–10, low to high quality) and the reference standard values for the test dataset of image slides (combined ProMPT/contemporary).

A sample WSI from the ProMPT cohort with the detailed quality overlays is presented in Fig. 2A. A heatmap is generated for the overall usability of each WSI at a patch-level, providing a simple visual cue as to the quality of regions of the image, clearly indicating poor quality areas for further assessment. The quality heatmaps are colour-coded to indicate the probability of a feature being present. As seen in Fig. 2A the heatmap indicates that the probability of most of the patches being ‘usable’ is high, with lower probability of staining issues, folding and other artefacts. The heatmap for ‘focus’ however indicates more patches predicted to have focus-related quality issue (note that here the white colour indicates slight while blue indicates severe focus issues), although correlating this with the ‘usability’ heatmap it can be seen that these issues do not invariably impact overall diagnostic ‘usability’. Thus, by comparing the overall usability heatmap with the individual heatmaps (indicate the various specific quality measures analysed) it is then possible to identify precisely which artefact(s) impact on the overall quality, and then assess whether an intervention, such as re-scanning, would improve the image. Heatmaps such as these could be integrated into a research pipeline and used to highlight artefact regions within a WSI for further assessment.

The performance of the proposed multivariate model on the test dataset of image patches is reported in Fig. 2B,C. The ROC-AUC for out of focus and staining artefacts is 0.85 and 0.84 respectively. However, as reported in Fig. 2B, the ROC-AUC scores for detection of focus and stain artefacts when these are severe, and therefore likely significant in relation to usability, indicate a higher model accuracy for such cases (0.99 and 0.97 respectively). This is likely a reflection of greater inter- and intraobserver agreement for severe artefact, in comparison with what was interpreted as a ‘slight’ artefact, with many of the latter cases showing minimal focus/staining artefact which in fact really fall on the borderline with “no artefact”.

A summary of predictions for the test dataset of image patches (combined ProMPT and contemporary) versus the reference standard (pathologist assessment), is shown in Fig. 2C. This shows that the average model predictions of the presence or absence of artefact, and for the ‘usability’ of an image, closely matches the subjective assessment by the pathologist. We then utilised the validated model to predict the average patch-level quality scores of the selected ProMPT cohort and the contemporary cohort separately (Fig. 2D). The results indicate that the average usability of image patches is predicted to be 93% for the selected ProMPT cohort and 97% for the contemporary (newer) cohort. This is in agreement with the intuition that quality issues might be more expected in older cohorts.

#### Slide-level quality assessment

A predicted QA at slide-level can be achieved by summarising the assessment made at patch-level. Whilst such a summarised QA is useful to identify dominant artefacts in a WSI, and thus those likely to impact on usability, it does not express a standardised scoring system. We therefore sought to fit a simple model to map the predicted patch-level measures to the subjective quality scores (0–10, from low to high quality) in accordance with the reference scores provided by an expert pathologist.

The slide-level subjective quality scores include a binary usability score of 0 or 1, and a standardised H&E staining score and a focus score, both from 0 to 10 (low quality to high quality, as previously). The dataset including whole slide subjective quality scores, was split into 60% train and 40% test with stratification based on the quality scores. Three separate linear regression models were then fitted to predict WSI usability, focus and staining scores from the mean and variance of selected quality overlays.

Reference standard subjective scores implied that 9% of WSIs in the selected ProMPT cohort (107 slides) and 2% of WSIs in the contemporary cohort (91 slides) were unusable, whereas our model predicted this to be 12% and 2%, respectively. Such slide-level analysis therefore implies that the quality/usability of WSIs in the ProMPT cohort is overall less than that of the contemporary cohort of cases, which is in keeping with the perception of the pathologists regarding the cohorts overall.

The predicted slide-level subjective scores can then be used to provide summary metrics for the whole cohort, including both the percentage of usable/unusable slides, and a histogram of WSI quality scores. As shown in Fig. 2E, our model-predicted assessment of scores for the selected slides of ProMPT and contemporary cohorts are highly comparable with the subjective pathologist assessment of the WSIs.

The Pearson correlation coefficient and the ROC-AUC of the predicted usability, focus and staining scores (versus reference standard) for the combined test set of slides is reported in Fig. 2F. This shows that the quality measures as predicted by PathProfiler closely align with the reference standard. The calculated accuracy of binary focus and staining scores with the cut-off $<= 4$, is 1, and the accuracy of the predicted usability score is 0.987.

### Non-prostate tissue cohort

Patch-level predictions of quality measures for all patches extracted from the 788 WSIs from the Fluorouracil, Oxaliplatin, CPT11 [irinotecan]: Use and Sequencing (FOCUS) cohort are presented in Fig. 1A. As for TCGA, many patches from this cohort are affected by ‘ink’, categorised as ‘other’ artefacts in our pipeline. Slide-level usability and standardised focus and staining quality scores for this cohort have been calculated for tumour regions only (Fig. 1B). Our model predicts that 86% of WSIs and 67% of image patches in the assessed FOCUS cohort are ‘usable’ according to our criteria (Fig. 1A,B).

**Figure 3:**
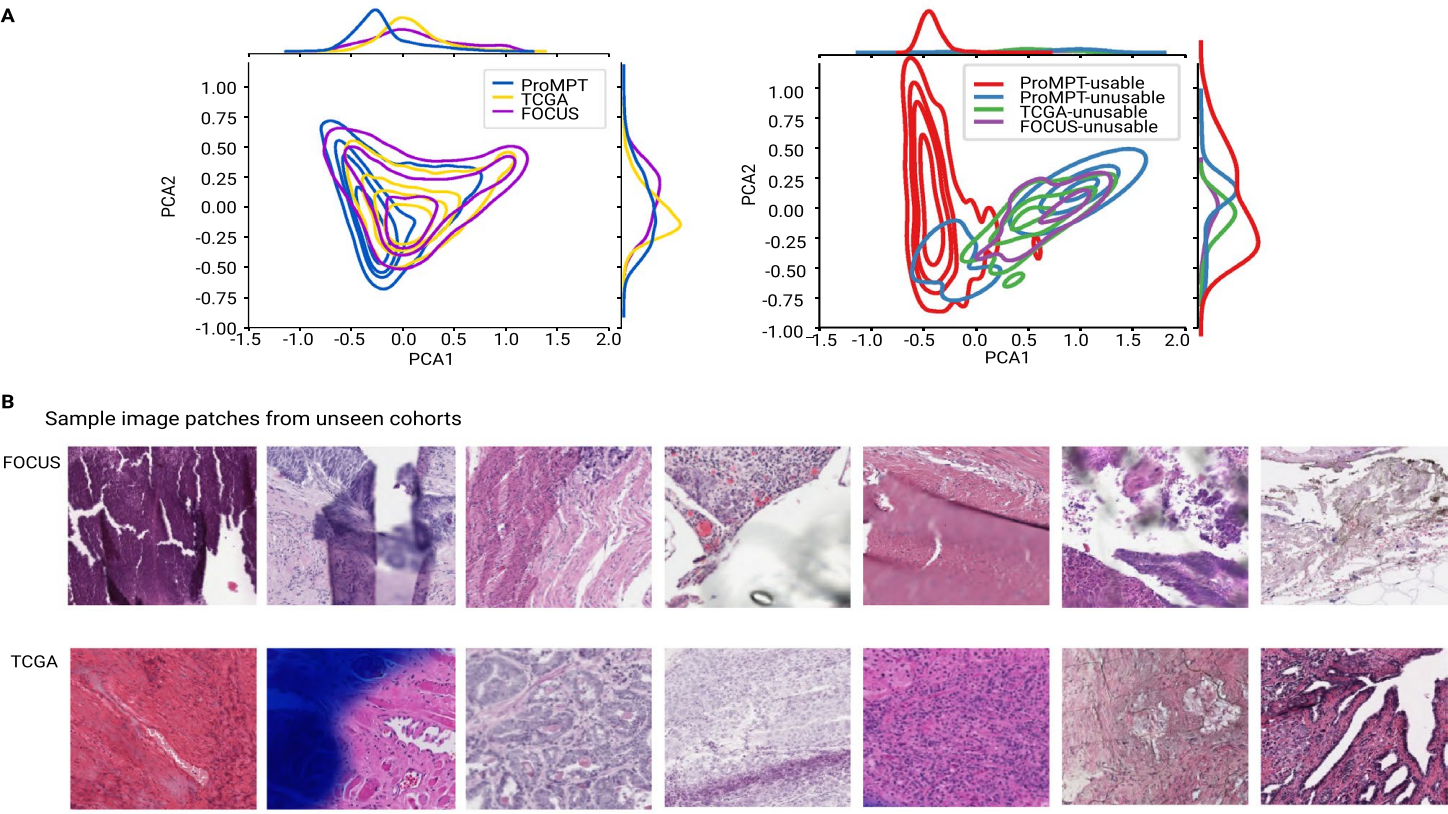
(**A**) Left: Kernel Density Estimation (KDE) plot of 2D PCA feature space for 16000 random patches extracted from ProMPT, TCGA and FOCUS cohorts. Whilst there is still room for improvement, the overlap between feature spaces of different cohorts suggests that we have a reasonable domain-invariant set of features. Right: Feature space of annotated patches in our patch test set from ProMPT cohort vs a set of annotated patches from TCGA and FOCUS cohorts. (**B**) Sample image patches predicted by PathProfiler as unusable from non-prostate tissue (FOCUS colorectal tissue) and TCGA (prostate tissue) cohorts.

Whilst we have trialed our QA pipeline to provide a quality estimate for an external prostate tissue cohort (TCGA) and non-prostate tissue cohort (FOCUS), using the same measures as we have assessed for the local ProMPT and contemporary prostate WSI cohorts, we must caveat that the model has not been trained on external prostate tissue WSIs nor on non-prostate tissues, both of which may harbour novel artefacts that the model has not been exposed to. For this reason, we do not claim that the results presented are fully reliable for these other cohorts. However, we have taken measures to assess for the presence of bias which may impact on the results, and we have shown that there is a good overlap between the feature space of the different cohorts, as shown in Fig. 3A.

Figure 3A (left image) illustrates a 2D PCA feature space of 16000 patches randomly extracted from each of the ProMPT, TCGA, and FOCUS cohorts. We also selected a small set of patches (66 from TCGA and 37 from FOCUS) predicted as unusable, some of which are included as examples in Fig. 3B. We then plotted the feature space of the unusable patches versus a set of usable and unusable patches from the ProMPT cohort. As seen in Fig. 3A (right image), the feature space of unusable image patches from all cohorts has a good overlap, suggesting that we have a set of features that are less biased to our cohort and maybe applicable to other cohorts to some extent. In our opinion therefore we have observed that PathProfiler-predicted quality measures for usability, focus, and other artefacts seems to provide a meaningful overall QA of other cohorts.

### Comparison with other quality assessment tools

We used our annotated dataset of image patches at $10\times$ and a set of hand-crafted features exploited in HistoQC to generate separate Random Forest classifiers to identify usability and each of the artefacts. Features used from HistoQC include TenenGrad contrast, RMS contrast, Michelson contrast, grayscale image mean, median and variance, mean of filtered image with Gaussian, Laplace, Frangi and Gabor filters, Local binary patterns (LBP), per channel mean of RGB and HSV and deconvolved H&E stain channels (available at https://github.com/choosehappy/HistoQC).

**Figure 4:**
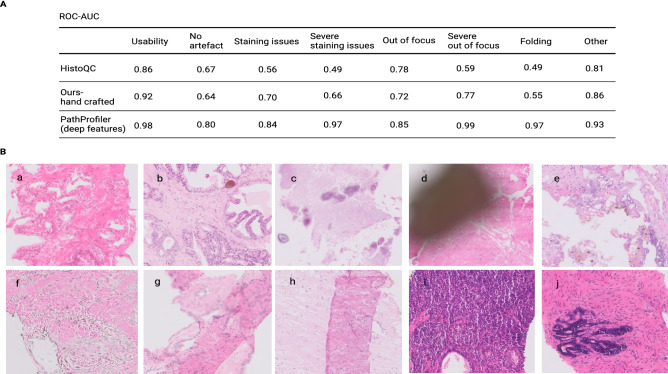
(**A**) QA of our annotated patch dataset (from ProMPT and contemporary cohorts) using other quality tools. (**A**) Comparison of performances of HistoQC, our proposed set of hand-crafted features, and PathProfiler for usability and artefact classification. (**B**) Examples of sample artefacts that are challenging to identify using hand-crafted features; (a, b) staining issues cannot be generally related to the brightness of images, (c, d) ‘other’ artefacts such as calcification (c) and dirt (d) that cause regions of low intensity variation are confused with out-of-focus regions. This may falsely reinforce recommendation by the algorithm for a slide re-scan, although this will not resolve those artefacts, (e, f) simple hand-crafted features such as Laplacian filtering misclassify unusable regions that contain rapid intensity changes as usable, (g, h) hand crafted features mostly associate folded tissue with darker colours in the data and therefore cannot detect folded areas within the range of average data colours, and (i, j) thicker tissue in a section may be misclassified as folded tissue.

We also tried a different set of hand-crafted features that performed better overall on our dataset in comparison with HistoQC feature set, as shown in Fig. 4A (denoted ‘Ours-hand crafted’). The features include Modified Laplacian (LAP2)^27^ focus measure, Variance of Laplacian, TenenGrad contrast, average difference of image from Gaussian filtered image, average variance along R, G and B channels (along third axis), per channel mean of RGB, HSV and deconvolved H&E stain channels and Otsu threshold. The ROC-AUC of classifiers generated using HistoQC features and our set of hand-crafted features versus PathProfiler are reported in Fig. 4A. As seen, the performance of our multi-task deep learning model in PathProfiler was superior to classifiers that used HistoQC or our ‘hand-crafted’ features.

**Figure 5:**
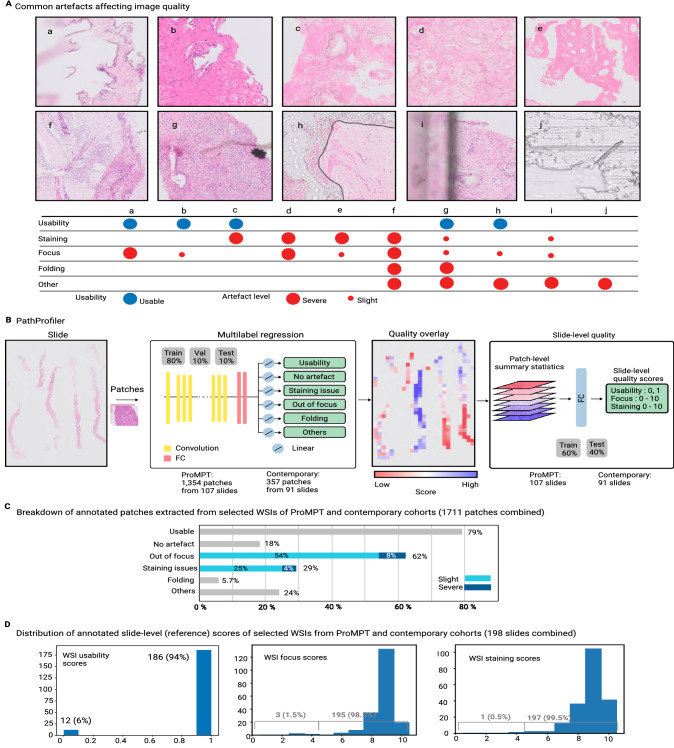
(**A**) H&E images demonstrating commonly encountered artefacts affecting digital WSI quality. Image quality may be affected by multiple artefacts, or just one, and focus quality can be specifically related to scanning/focus issues or it can be a result of other artefacts. (a, b) focus issue, (c) fading/loss of contrast of H&E stain, (d, e) both staining and focus issues (f, g) a combination of focus and staining issues with tissue folding and dirt, (h) bubble under the coverslip and slight focus issues in visible tissue area, (i) edge of the coverslip (affecting focus), (j) coverslip glue. (**B**) PathProfiler QA pipeline. After tissue segmentation, patches of $256 \times 256$ are extracted at $5\times$ magnification and resized to $224 \times 224$ to accommodate for the ResNet18 CNN model. For each patch, the trained model predicts the presence of an artefact, and for focus and H&E staining artefacts it also predicts a quality score for each patch, 0 = no quality issue, 0.5 = slight quality issue, 1.0 = severe quality issue. A quality overlay is generated for each output category. In the next step, we map the predicted quality overlays to the slide-level standardised scoring system. For this, statistical parameters of quality overlays are used to predict slide-level quality scores; overall usability of the WSI (binary 0 or 1), and a score 0–10 for quality of focus and H&E staining from the lowest quality to highest quality, where the cut-off score for acceptable quality for diagnostic purposes is 4. (**C**) The composition of the pathologist-annotated image patches extracted from selected WSIs of ProMPT and contemporary cohorts (combined) (**D**) The distribution of pathologist-annotated (reference) quality scores of WSIs selected from ProMPT and contemporary cohorts (combined) for WSI usability (binary 0 or 1), focus and H&E staining quality (0–10, as above).

We found multiple challenges in using hand-crafted features for our prostate specimen cohorts, including those previously proposed by Janowczyk et al.^26^. For example, H&E staining issues detected in the ProMPT cohort cannot be directly correlated with image brightness, and we found that overall the available hand-crafted features were not successful in the detection of various staining issues, such as those illustrated in Figs. 4B (a,b) and 5A (c–f).

Furthermore, many available QA tools measure the intensity variation in an image (e.g. using edge-detection filters) to identify blurred or unusable regions^16,23,26^. We found that this resulted in large gland-free stromal regions in our data being designated as ‘unusable’, an issue also previously recognised by Janowczyk et al.^26^.

We faced further challenges; artefacts that result in reduced variation in colour or intensity, such as H&E poor staining Figs. 5A (c–e) and 4B (a), cover slip edges Fig. 5A (i), calcification and ink/dirt Fig. 4B (c,d) are confused with out-of-focus regions. This is undesirable as this will incorrectly identify the slide as needing a rescan, which will not resolve the quality issue. On the other hand, unusable regions that contain rapid intensity changes such as Fig. 4B (e,f) were detected as usable with hand-crafted features.

Finally, we found that the hand-crafted features dramatically failed in the detection of folded tissue in our dataset. This would appear to be the result of the association of tissue folding with darker (H&E) colours^26^ while it rather seems to be recognisable by more complicated features (Fig. 4A). For example, hand-crafted features failed in the detection of folded regions in Fig. 4B (g,h) and Fig. 5A (f) as the colour intensities in these images are generally in the range of average colour values in the dataset. On the other hand, areas where the tissue section was thick and the H&E consequently appeared dark, such as in Fig. 4B (i,j), were misclassified as folded tissue.

## Discussion

Through deep-learning we have developed an AI model to automate the assessment of WSIs for quality related measures, using a subjective cut-off threshold for diagnostic usability of a WSI, as assessed by an expert pathologist, as being the minimum acceptable quality standard for a WSI for computational pathology. We set out to achieve this utilising the ProMPT cohort as a representative and academically important example of a historic glass slide cohort. In so doing, we have developed a pipeline, designated as ‘PathProfiler’, for QA of WSIs at both patch-level and slide-level. PathProfiler facilitates the identification of quality-related issues in the form of a heatmap (Fig. 2A), providing focus for direct visualisation of problematic areas of an image in an attempt to determine whether a specific intervention such as re-scanning or re-staining (H&E) of a glass slide could be of benefit in improving the image quality. Predicted patch-level quality scores are assimilated to three slide-level scores for usability, focus and staining quality. In addition, PathProfiler offers the opportunity to identify and exclude low-quality patches from assessment, negating the need to render a whole image as unsuitable on a quality basis. A quality control tool such as PathProfiler could then be utilized to further investigate how low-quality data or pathologist’s subjective QA affect AI decisions.

Whilst PathProfiler is developed on local prostate tissue cohorts including ProMPT, we have also attempted to demonstrate its functionality on other tissue cohorts. Finally, we have assessed the functionality of PathProfiler in comparison with other available QA methods.

Intuitively, quality-related issues might be expected to be more frequently encountered in cohorts of older slides due to degradation, and whilst this is the anecdotal experience of histopathologists, to-date there is little evidence to support such an assumption. We therefore sought such evidence at the outset of our study by investigating the frequency of significant quality issues, as judged by a specialist pathologist on the basis of whether an image was usable for diagnostic purposes. We compared the findings within the WSIs of a cohort of historic glass slides with those from a newer ‘contemporary’ cohort. As an example of an historic cohort, we had access to the collection of almost 4000 glass H&E slides of prostate tissue (mainly biopsies) from the ProMPT observational study, which were collated between 2001 and 2018, and retrospectively digitised. For the purpose of algorithm development, a representative set of WSIs from 10% of cases (107 WSIs) from this cohort were subjectively assessed for quality related features and overall diagnostic usability, and 9% of these were considered to be ‘unusable’ in their current state. This compared with 2% of cases from a comparably sized contemporary cohort of WSIs of prostate biopsies from 2019, scanned at the time of slide preparation. These figures may be an underestimate of quality given that they are based upon the requirement for subjective agreement on usability from all three participating specialist pathologists, however for algorithm development purposes it was felt that capture of all potential quality issues would be more beneficial at the outset. From the annotated WSIs it appears that the impact on ‘usability’ is likely to be attributable to more significant focus quality issues seen in the ProMPT cohort compared with the newer cohort, although this subjective assessment indicated that the quality of H&E staining was also an issue more frequently seen in the ProMPT WSIs, as might be expected given the recognition that H&E staining fades over time. These glass slides from both cohorts had been prepared at the same institution and whilst there may have been minor variation in H&E preparation given the period over which they were produced, this would be expected to be minimal, and therefore any differences can reasonably be considered to be related to slide age. What became apparent also was that the un-usability of images was not necessarily related to a scanning issue per se; other intrinsic tissue-related features such as folding and other artefacts such as cover slip edge, dirt and calcifications also impacted on usability (sample images and their QA are shown in Fig. 5A). This was important to recognise at the outset as we sought to develop a pipeline that could predict not only the presence of a significant quality related issue, but one that could potentially be remedied by rescanning or restaining the tissue section. The ability of the model to both detect and classify quality issues that could not be remedied was therefore important in ensuring the accuracy of the tool in this task.

The recognition of considerable staining issues in retrospective cohorts calls into question the effectiveness of traditional colour normalization techniques^28^ as a preprocessing step for image analysis or AI algorithms. Based on our observation with the ProMPT cohort, staining issues are mostly related to the loss of colour detail in the nucleus. In severe cases, there was almost no colour variation across the H&E image with a loss of contrast; nucleus vs cytoplasm vs stroma, e.g. image patch (e) in Fig. 5A. While advanced normalization techniques, e.g. using generative models^29^, could be of interest in severe cases, simple transformation techniques may fail to adapt.

At both patch-level and slide-level for these prostate cohorts, PathProfiler is able to accurately predict the usability of an image, with an ROC-AUC at slide-level of 0.987 (compared with the reference standard pathologist assessment, Fig. 2F). At slide-level the ROC-AUC for focus and H&E staining overall is 0.826 and 0.751 respectively. At patch-level, if such evaluation is limited to severe artefact, which is of more significance in terms of usability, the ROC-AUC is improved from 0.85 to 0.99 and from 0.84 to 0.97, respectively (Last table row in Fig. 4). As such, PathProfiler predicts that in overall 7.5% of WSIs (12% of the selected ProMPT cohort (107 WSI) and 2% of the contemporary cohort (91 WSI)) are ‘unusable’ at a diagnostic threshold, which is very close to the reference standard 6% (9% and 2% respectively). PathProfiler flagged all slides with focus and staining scores less than 5 (1.5% and 0.5% of slides respectively), reported as accuracy=1 with threshold 5 in Fig. 2F. It should be noted that the cut-off threshold from the predicted scores was at a level at which interobserver agreement may not be high, i.e. at the mid-range of the scale (Fig. 2E). In reality, predicted values are from a continuous range (0 to 1 for usability, and 0 to 10 for focus and staining scores) and therefore the cut-off quality thresholds could be shifted in accordance with local preference, and feasibly such a preference could also be impacted by variable quality requirements conferred by differing tissue types and diagnostic questions. We also observed that the performance of patch-level model predictions is higher for ‘severe’ artefact, in comparison with what was interpreted as a ‘slight’ artefact (ROC-AUC of 0.99 vs 0.85 for severe vs slight focus issues, and 0.97 vs 0.84 for staining issues). This is likely a reflection of lower inter- and intraobserver agreement for ‘slight’ artefact detection, with many of such cases showing minimal focus/staining artefact which in fact really fall on the borderline with “no artefact”.

The PathProfiler assessment of the same quality measures across the entirety of the ProMPT cohort (3819 WSI), demonstrated a predicted usability of 89% of the WSIs (at slide level); a figure aligned with that expected on the basis of the original subjectively assessed WSI cohort, and with the perception of the pathologists working with the cohort. This is perhaps a useful benchmark for expectations for similar single-institutional historic cohorts, although this is unproven. Potentially more usefully, PathProfiler predicts that at least 2% of the ProMPT cohort (84 slides) could see quality improvement from re-scanning, and at least 0.45% (17 slides) from re-staining. Such relatively minor interventions would be worthwhile in such a precious cohort, and anecdotally we have certainly seen improvements in image quality from slides in this cohort which have been re-stained. Furthermore, specifically in relation to prostate biopsy cases, even if the originally scanned H&E WSI is eventually deemed unusable, the routine availability of multiple H&E levels on a block, often put onto different H&E glass slides, offers the potential opportunity to scan alternative slides from the same case.

The ProMPT prostate cohort was considered at the outset to be representative of other retrospective historic cohorts in terms of quality in that it was a non-curated resource at the time of scanning. However, we wished to investigate the functionality of PathProfiler on other prostate tissue cohorts, and for this purpose we identified the TCGA prostate cohort, being an example of a publicly available multi-institutional dataset that is already in use for computational pathology. We also applied PathProfiler to a non-prostate tissue cohort, the MRC FOCUS colorectal cancer dataset. These additional assessments were undertaken with caution given that features associated with image QA can vary widely among different cohorts due to inherent tissue characteristics, differences in tissue handling (e.g. section thickness) and preparation (formalin-fixed paraffin embedded (FFPE) versus frozen section), and to differences in digitisation of the WSI not only associated with digital system used. This was potentially of particular significance in relation to the TCGA dataset, wherein most of the tissue sections are from frozen rather than FFPE tissue. While there are techniques to reduce domain-related biases in algorithm development, such as data augmentation, they cannot be freely used for learning features that describe image quality, due mainly to the fact that many of these techniques change the quality of an image to augment the data, which would be undesirable for a study of inherent WSI quality. For example, transfer functions that change colour saturation, contrast and brightness, add structures or noise, or use filters such as Gaussian blur would not be recommended in this context.

Whilst we therefore have trialed PathProfiler on the cohorts that are different from ProMPT, we do not claim that the results are fully reliable on those cohorts, although the demonstration of overlap of feature space for the different cohorts (ProMPT, TCGA, FOCUS, Fig. 3A) provides reassurance of a domain invariant set of features for our model. Within this context, PathProfiler does appear to provide meaningful predicted measures for focus, usability and other artefacts for an overall QA of other cohorts, and predicts 90% of WSIs in the TCGA cohort, and 86% of WSIs in the FOCUS dataset to be usable according to our criteria Fig. 1B. Perhaps significantly it has also been revealed through patch level scores that the ‘other’ artefact category appears to be responsible for many of the ‘unusable’ areas on the TCGA cohort (Fig. 1A), which relates to the presence of ink. By comparison, the ink was removed prior to the digitisation of the ProMPT cohort which may go some way to account for the quality differences between cohorts, although this is supposition. Ink^30^ is unlikely to be an issue in prospectively digitised images if these are produced within a routine digital pathology laboratory diagnostic workflow.

Comparing this model with other available tools seemed the intuitive next step, and for the purpose of comparing a tool with the ability to assess a range of artefacts we selected HistoQC^26^ which uses a set of hand-crafted features for supervised classification. There were challenges with this approach, in particular in relation to classification of issues associated with H&E staining quality and tissue folding. We found that these methods were not satisfactory in the classification of the range of artefacts that impacted on image usability, being unable to separate factors that could be remedied by re-scanning or re-staining from those that could not. For instance, our proposed model is more reliable in distinguishing out-of-focus regions from glue or ink (indicated as other category). As a result, our deep learning model overall appears superior in performance, although we recognise that our datasets for testing are relatively small.

Further development of PathProfiler would therefore seem to be potentially worthwhile, and tools such as this will likely become increasingly important; as AI algorithms become available for use within the diagnostic setting, the quality assurance related to their development will likely be scrutinised^13,31^, much in the same way as for other medical devices. It is foreseeable that there will be a need to provide evidence of the quality of cohorts used for the development of AI tools being considered for clinical use, or of a means to evidence that quality-related issues had been considered and accounted for during the study design. Ideally, a quality indicator could be provided alongside a WSI library to inform potential researchers of the basic quality of the cohort and the nature of artefacts present. However, in our opinion the development of such a tool capable of analysis across tissue types now requires a concerted effort to collate larger and more varied sample cohorts to enable exposure to the full range of potential variables, ensuring reliability and lack of bias. As such, we have made the code, trained models and generated quality overlays available at https://www.github.com/MaryamHaghighat/PathProfiler for further studies.

As digital pathology is rolled out within the diagnostic setting, a quality tool such as PathProfiler will have future potential within a clinical workflow. The pipeline is optimized for speed and memory and is implemented efficiently for both CPU and GPU devices. PathProfiler works on relatively low magnification, enabling the tool to run at speeds that will not impact the throughput of images which would otherwise delay turnaround times. The reported throughput of QA only is $89.13 \pm 24.22$ tiles/s on Nvidia Quadro RTX 6000 GPU, therefore an average prostate biopsy slide in our cohort would take around 10 s to run through the QA. A quality tool integrated into the workflow offers potential to improve efficiency by indicating suboptimal WSI in real time. It may also assist technical improvements within the laboratory through consistent flagging of quality issues such as tissue folding, poor staining, that may otherwise be more subjectively reported by users on a more ad hoc basis. It must however be considered before deploying an AI tool into a diagnostic setting whether the relevant regulatory and accreditation requirements have been satisfied. PathProfiler, in current form does not have a formal regulatory clearance for diagnostic use.

Whilst the benefit for a clinical workflow in highlighting sub-optimal WSIs is evident, there is also a downstream benefit for research, and increasingly so. Retrospective cohorts of cases have been the mainstay of data resources to date for computational pathology, and for research requiring long-term patient outcome data they will remain so, however, the roll-out of digital pathology within the diagnostic setting has opened the doors for prospective collation of whole slide images (WSI) for research purposes. As a result, resources are being ploughed into forward-looking schemes which involve the prospective collation of WSI directly from the diagnostic workflow. The Pathology image data Lake for Analytics, Knowledge and Education (PathLAKE) project (https://www.pathlake.org) is an exemplar of such a scheme drawing on WSIs from multiple academic clinical settings to populate a repository that can be utilised for algorithm training, in a pioneering partnership between industry and the NHS. Whilst it is anticipated that these contemporary diagnostic slides would be afflicted with fewer quality issues and we have seen this in our old vs new data (12% vs 2% unusable slides), artefacts are still recognised and quality assurance is still very relevant.

As part of future work, we will optimise the model to be deployed as part of the data acquisition effort. Our annotated data size is relatively small and does not cover various artefacts seen in different cohorts. We are aiming to improve the tool performance on other cohorts by aggregating more data. This can be done through semi-supervised learning methods and information extracted from diagnostic reports to limit input required from experts. Furthermore, with the help of the community to collect various artefacts in different tissue types and settings, we believe PathProfiler can be extended to a comprehensive and clinically relevant QA tool.

The employed CNN architecture in this work was chosen based on available resources. Further investigation is required to find an optimal network architecture and hyper-parameters.

## Methods

The size and complexity of our prostate cancer cohort which motivated this work is described in “Prostate cancer cohort selection”. The rationale for developing PathProfiler is discussed in “PathProfiler-a pipeline for comprehensive quality assessment”. The application of the tool to our prostate cancer cohort, prostate cancer slides from TCGA, and other histology cohorts, is presented in “Quality assessment of cohorts”. We selected HistoQC^26^ to benchmark PathProfiler. While other quality tools are available, they typically only assess specific artefacts as for example quality of focus. HistoQC exploits a set of hand-crafted features to train supervised classifiers. The challenges of hand-crafted features for QA of our prostate specimen cohort are discussed in “Comparison with other quality assessment tools”.

### Prostate cancer cohort selection

We utilised two cohorts of H&E slides from formalin-fixed paraffin embedded (FFPE) prostate tissue for algorithm development: a retrospective cohort of historic slides from the ProMPT study, and a ‘contemporary’ cohort from the routine diagnostic workflow. These included biopsies and transurethral resection specimens from the prostate (TURP).

The ProMPT cohort comprises 4732 histology slides (3819 H&Es and the remainder immunohistochemistry) collated between 2001 and 2018 as part of a UK-based observational study in which participants underwent routine histological assessment as part of diagnostic pathways. All slides digitised for this study were from one site (the lead site—a large academic teaching hospital) which stores slides older than 2 years in an off-site accredited archival storage facility. The combination of the clinical outcome data together with the histological images for this cohort makes it a rare and highly significant resource for image analysis-based research, whilst also providing a relevant example cohort for the purpose of this study.

The ProMPT slides were retrospectively digitised during the period 2017–2021 and are therefore considered representative of a historic glass slide cohort. The collection includes predominantly prostate biopsies, together with transurethral resection specimens from the prostate (TURPs), and radical prostatectomies (RPs). In the initial stages of the digitisation process, it was recognised that there were quality issues with the scanned images of a small proportion of the cases that might potentially have an impact on downstream image analysis; features considered common to other historic cohorts. These included out-of-focus regions, variability in H&E staining quality (fading/loss of contrast), tissue folding, air/glue ‘bubbles’ under the coverslip, dirt, pigments, cover slip edge, and surgical diathermy (such as illustrated in Fig. 5A).

For training the model, a random 10% of cases (glass slides from 2008 to 2016) from the ProMPT cohort were identified from which we selected one H&E WSI (the first available). There were in total 107 WSI from H&E slides, representing 99 biopsies and 8 TURPs.

For quality comparison and enhancing the training dataset with a contemporary cohort, the first available H&E WSI was selected from a set of 91 consecutive prostate biopsy cases from the contemporary diagnostic workflow which had appropriate patient consent. These slides had been scanned immediately after being generated as part of the diagnostic workflow.

The H&E slides from both cohorts were produced and scanned in the Cellular Pathology Department at Oxford University Hospitals NHS Foundation Trust (OUHFT), scanning on a Philips UFS scanner.

The retrospective study was conducted under the ProMPT ethics (reference MREC 01/4/61). The prospective study was conducted under the Pathology image data Lake for Analytics, Knowledge and Education (PathLAKE) research ethics committee approval (reference 19/SC/0363) and Oxford Radcliffe Biobank research ethics committee approval (reference 19/SC/0173). Patients were not identifiable from the material. The research was performed in accordance with the Declaration of Helsinki. Informed consent was obtained from all subjects and/or their legal guardian(s).

### PathProfiler-a pipeline for comprehensive quality assessment

Working with large cohorts requires an automated method for assessing whether a WSI (or a region of a WSI) is usable or not at the diagnostic level, with the assumption that an image of appropriate quality for diagnosis would be suitable for downstream computational pathology, including algorithm development, with diagnostically unusable images considered inappropriate for such use. If a WSI is considered unusable, then ideally there should be an indication as to whether an intervention, namely re-scanning or re-staining (then re-scanning) could potentially resolve the quality issue that had been detected. This would require also the categorisation of the WSI in terms of common artefacts present, and separately, where appropriate, an indication of the severity of the artefact (e.g. see table in Fig. 5A).

We thereby designed PathProfiler (illustrated in Fig. 5B) to indicate simultaneously the ‘usability’ of an image and the presence of artefacts. In this way, the algorithm can predict if a WSI is unusable, but also if it is associated only with severe issues with either focus or H&E staining. This facilitates the identification of the image within the pipeline for consideration of re-scanning or re-staining in order to improve the quality and render it ‘usable’. Alternatively, this approach facilitates the user to identify when re-scanning or re-staining would not resolve the quality issue such as when presented with data for a WSI with a significant number of intrinsic artefacts, dirt, ink or bubbles in the tissue area.

Firstly, a tissue segmentation model^32^ extracts tissue regions. The current version of the algorithm requires patches of $256 \times 256$ at $5\times$ magnification as input. Extracted patches are resized to $224 \times 224$ to accommodate for a ResNet18 CNN model. A multi-label pre-trained model predicts a set of quality measures for each patch, which includes the H&E staining and image focus, both indicated by a three-level score; 0 = no quality issue, 0.5 = slight quality issue, 1.0 = severe quality issue. This models the subjective pathologist assessment, whereby a score of 0.5 is assigned for a quality issue felt to be minimal and insufficient to render the WSI unusable, whereas a score of 1 implies severe artefact, potentially (but not necessarily) rendering the WSI unusable. Hence the predicted staining and focus scores can be interpreted as the severity of the artefact. The model also predicts the presence of additional artefacts, categorised as ‘tissue folding’ or ’other’, the latter including dirt, diathermy artefact, etc, although these are not associated with a severity ‘score’.

The predicted patch-level quality measures are collated to generate a WSI quality overlay for each category. Using statistical parameters of the quality overlays, we predict the subjective quality scores (i.e. those expected from an expert pathologist) at slide-level. These slide-level quality scores include overall usability of the WSI (binary 0 or 1), and a score from 0 (lowest quality) to 10 (highest quality) that predicts the overall quality of H&E staining and of image focus. In our study this score was in accordance with a 10-point UKAS-approved qualitative scoring scale utilised within the Cellular Pathology Department for assessment of H&E quality on glass slides. According to this system, a score of $<=4$ is considered a ‘fail’ for quality, 5–6 = pass, 7–8 = acceptable (good) level of quality, 9–10 = excellent (high) level of quality. With this information it is possible to predict the pathologists’ subjective quality scores indicating whether the WSI should be accepted as being of appropriate diagnostic quality, or rejected, and this is also considered indicative of the potential need for re-scanning or re-staining.

#### Data annotation

A subset of our prostate cancer cohort was annotated by specialist urological pathologists to facilitate training and testing. As described above, this dataset comprised 107 H&E stained WSIs of prostate tissue (biopsies and TURPs) from the ProMPT cohort and 91 H&E stained WSIs of contemporary prostate biopsy cases from the contemporary diagnostic archive. The selected slides provided a dataset of 1711 annotated image patches which was divided into the training set (80%), test set (10%), and validation set (10%), and a dataset of 198 annotated whole slides which was divided into the training set (60%) and test set (40%).

The breakdown of the annotated dataset of image patches is shown in Fig. 5C, and the distributions of the annotated dataset of image slides for usability, focus and H&E staining is shown in Fig. 5D.

Image patches were manually selected for annotation to cover various combinations of artefacts such as focus or H&E staining quality issues, tissue folding, dirt, and damage due to diathermy. While the pipeline used image patches of $256 \times 256$ at $5\times$, for the annotation task during development, we opted to use patches of $512 \times 512$ at $10\times$ magnification as this was considered by the pathologists to be more representative of the way in which the WSI would be viewed in the diagnostic setting, thus facilitating more realistic images for subjective scoring. The area for pathologist annotation was a designated highlighted area of $712 \times 1024$ pixels within a $512 \times 512$ box in the centre of the image patch; providing the highlighted image within the larger patch for assessment was considered to provide a context more representative of the diagnostic setting. The annotation task was then performed by the pathologist using a digital pathology workstation clinically validated for diagnostic work. For each image the pathologist provided quality data within a drop-down menu in terms of the overall usability of the image patch (for diagnostic interpretation), the presence and severity of focus and/or H&E contrast issues, the presence or absence of additional specific artefacts; folding or other artefacts. The corresponding labels are listed in Table 1. Subsequently, a multi-label target ${\mathcal {Y}}=[ y_1,\ldots , y_6 ]$ was associated with each image patch. The composition of our annotated (combined ProMPT and contemporary cohort) multi-label patch dataset is illustrated in Fig. 5C.

**Table 1:** Patch level labels. The proposed annotation protocol captures that a given image patch can be corrupted by multiple different artefacts. This protocol also captures if an image patch is diagnostically usable despite being affected by some artefacts.

Label	Criteria	Value
$y_1$	Usability	1—appropriate for diagnosis
		0—otherwise
$y_2$	No artefact	1—no presence of any slight or severe artefacts
		0—otherwise
$y_3$	Staining artefacts	1—severe staining or H&E contrast issues
		0.5—slight staining or H&E contrast issues
		0—no staining or contrast issues
$y_4$	Focus artefacts	1—severe focus artefacts
		0.5—slight focus artefacts
		0—no focus artefacts
$y_5$	Tissue folding	1—the presence of tissue folding
		0—otherwise
$y_6$	Other artefacts	1—the presence of other artefacts such as dirt, glue, ink, cover slip edge, diathermy, bubbles, calcification and tissue tearing
		0—otherwise

For whole slide QA, three specialist urological pathologists independently assessed the 198 WSIs from the combined ProMPT and contemporary dataset for: (1) overall usability—a binary label if the slide can be used for diagnosis, (2) focus quality, and (3) H&E staining quality.

Quality of focus and H&E staining assessment were standardised from 0 (lowest focus quality) to 10 (highest focus quality), utilising the qualitative scale discussed before. A score of $<= 4$ is considered a fail for quality, with 9/10 being equivalent to excellent quality. To aggregate individual assessments, the focus and staining quality scores were averaged between the three assessors. However, we used the “AND” logical operator to aggregate the diagnostic usability of slides such that a slide is rendered usable only if all three assessors considered it as being usable. Whilst this approach may increase the rate of false-positive for identifying unusable slides, it was preferred over missing such cases. The aggregated reference WSI quality scores from the ProMPT cohort included 97 (91%) usable and 10 (9%) unusable WSIs, and from the contemporary cohort 89 (98%) usable and 2 (2%) unusable WSIs. This subjective assessment mirrored the overall perception of the pathologists that the majority of WSIs in both cohorts were of suitable quality for diagnosis. The distribution of annotated quality-related scores of the combined WSIs (198) are shown in Fig. 5D.

#### Multi-label neural network training

In our multi-label dataset the *d*-dimensional input features in ${\mathcal {X}} = {\mathbb {R}}^{d}$ are associated with the multi-label target space ${\mathcal {Y}}=[y_1,\ldots , y_c]$ where $[y_3, y_4] \in \{0, 0.5, 1\}^{2}$ and $[y_1, y_2, y_5, y_6] \in \{0, 1\}^{4}$ with a total of $3^{2}\times 2^{4}=144$ possible combination of unique labels. So, the multi-label model learns a function $f: {\mathcal {X}} \rightarrow {\mathcal {Y}}$ from the training data ${\mathcal {D}} = \{ ({\mathcal {X}}_i, {\mathcal {Y}}_i ) \vert i=1,\ldots , m\}$.

Whilst the problem here could be treated as a combination of single regression or single binary classification tasks, we found it more efficient to use a multivariate regression model. First, a multi-label model can efficiently exploit correlations among labels^33^. Second, we ran multiple tests and observed that label noise in dataset preparation for studying quality was inevitable. This was mainly due to the inherent challenges in subjective QA, including both intraobserver and interobserver variation and in the definition of acceptable quality thresholds (for diagnosis), which again varied. We attempted to reduce the “noise” in this data through provision of guidance for standardisation of quality scoring (e.g. the UKAS approved 0–10 scale), and through regular discussion amongst the pathologists and the wider group to encourage consistency in interpretation of quality, with the provision of example images. The study took place over a prolonged period, making such standardisation important, to reduce the impact of noise. The pathologists used the same workstation throughout for assessment of WSI to minimise bias.

Strong classification loss functions do not always perform well under noisy labels^34^. The Binary Cross Entropy (BCE) loss function for instance highly penalises large loss values, leading to a model learning in favour of outliers rather than inliers. Whilst different approaches are proposed to efficiently learn from noisy data^35^, we employed the Huber loss function which is in the category of robust loss functions to label noise. It is shown that Huber loss can provide efficient prediction in deep learning under minimal assumptions on the data^34^. Huber loss function combines the benefits of mean squared loss for small loss values while rejecting the dominance of outliers similar to mean absolute loss for large loss values^34^.

For data augmentation, we arbitrary rotated patches ($0^{\circ }$, $90^{\circ }$, $180^{\circ }$, $270^{\circ }$) and flipped them along the vertical or horizontal axis. A random affine transform including shear from $-10^{\circ }$ to $10^{\circ }$ and translation by $-5$ to 5% per axis, were applied. In addition, we randomly perturbed the hue of images by a value from $-30$ to 30.

We trained an 18-layer deep ResNet^36^ model (ResNet18) which takes 3-channel input images at a size of $224 \times 224$ pixels. The last fully connected layer was modified to output six classes with linear activation functions. Multi-label classification was performed with a Huber loss with $\delta =1$, as shown in Eq. (1) and an Adam optimiser.

1 $$\begin{aligned} L_\delta (x, y) = {\left\{ \begin{array}{ll} \frac{1}{2}(x-y)^2 &{} \text {for } |x-y| \le \delta , \\ \delta \, |x-y| - \frac{1}{2}\delta ^2 &{} \text {otherwise.} \end{array}\right. } \end{aligned}$$

During training, we down-sampled annotated image patches of $512 \times 512$ at $10\times$ to the network $224 \times 224$ pixels input requirement. Therefore, we effectively evaluated slide quality at $5\times$ magnification.

Training with initialised weights, pre-trained on the ImageNet dataset, was performed with hyper-parameters of batch size = 100 and learning rate = 1e − 4. The dataset was split into three partitions for training (80%), testing (10%) and validation (10%) with stratification based on their unique multi-label combinations. We ran the model training for 200 epochs and selected the model based on minimum loss for the validation set. To partially handle the label imbalance, a weighted batch sampler was used.

### Quality assessment of cohorts

While the models were trained on annotated data from prostate slides we also tested the model on other cohorts to assess its generalisability. Firstly though we applied PathProfiler on the entirety of the available H&E stained WSIs (3819 WSIs) from the ProMPT cohort to provide a prediction of usability of the whole cohort, and to assess the burden of artefacts present within the cohort, seeking to potentially identify WSIs with quality issues that might be resolved.

To analyse the performance of our tool on other previously unseen WSI of prostate tissue, we selected slides from the TCGA multi-institutional collection. All 449 examples of H&E WSIs of prostate tissue in TCGA were analysed. These included mostly RP cases, and a range of preparations, including frozen tissue as well as the FFPE tissue, the latter more routinely used in clinical practice^37^. Noteworthy is that the focus of the tissue samples collected within the TCGA cohort is different to that of our cohorts, with samples collected primarily for molecular analysis, hence the predominance of H&Es from frozen sections in TCGA rather than FFPE tissue such as that present in our training/test cohort. As such we anticipated that the cohort would vary from that on which PathProfiler had been trained. The quality data sought from the analysis included the parameters assessed on our original cohorts, i.e. overall usability, focus and H&E staining quality, and presence of other artefacts, however the model provided estimates of these parameters rather than predictions as we did not have a reference standard pathologist QA for the TCGA WSIs.

Finally, WSIs from another cancer cohort were assessed to determine the functionality of our model on other tissue types. For this purpose we opted to utilise PathProfiler on the FOCUS cohort (Fluorouracil, Oxaliplatin, CPT11 [irinotecan]: Use and Sequencing), which is a collection of 788 WSIs from a dataset of colorectal cancer specimens taken from the MRC FOCUS clinical trial^38^. These were WSIs of resected tumour from 375 advanced colorectal cancer patients. Glass H&E slides from this cohort were reviewed by a specialist gastrointestinal pathologist; tumour and the associated intratumoural stroma were annotated and used to guide RNA and DNA extractions for the purpose of other studies. All H&E slides were later scanned (in 2016) at high resolution on an Aperio scanner at a total magnification of $20\times$. WSIs were then re-reviewed by a second gastrointestinal pathologist and tumour annotations were traced to generate region annotations for machine learning classification^39^.

The quality data sought from the analysis of the FOCUS cohort included the parameters assessed on our original prostate cohorts and the TCGA cohort. However, for FOCUS, slide-level scores were calculated based on tumour area only. It was considered more appropriate to limit the assessment to the tumour because there were large areas of tissue, such as fat, in many of the WSI that were not comparable with prostate tissue and we wanted to avoid this tissue skewing the data. However, at patch-level the average estimated quality scores were calculated for patches extracted from all tissue regions.

## Data Availability

The datasets generated during and/or analysed during the current study are not publicly available due to the terms of the PathLAKE Consortium Agreement and other agreements in place but a subset of the data could be made available via the corresponding author on reasonable request. The software is open source.
